# Prediction of Online Psychological Help-Seeking Behavior During the COVID-19 Pandemic: An Interpretable Machine Learning Method

**DOI:** 10.3389/fpubh.2022.814366

**Published:** 2022-03-03

**Authors:** Hui Liu, Lin Zhang, Weijun Wang, Yinghui Huang, Shen Li, Zhihong Ren, Zongkui Zhou

**Affiliations:** ^1^Key Laboratory of Adolescent Cyberpsychology and Behavior, Ministry of Education, Wuhan, China; ^2^Key Laboratory of Human Development and Mental Health of Hubei Province, Wuhan, China; ^3^School of Psychology, Central China Normal University, Wuhan, China

**Keywords:** prediction, online mental health service, COVID-19, online psychological help-seeking, interpretable machine learning

## Abstract

Online mental health service (OMHS) has been named as the best psychological assistance measure during the COVID-19 pandemic. An interpretable, accurate, and early prediction for the demand of OMHS is crucial to local governments and organizations which need to allocate and make the decision in mental health resources. The present study aimed to investigate the influence of the COVID-19 pandemic on the online psychological help-seeking (OPHS) behavior in the OMHS, then propose a machine learning model to predict and interpret the OPHS number in advance. The data was crawled from two Chinese OMHS platforms. Linguistic inquiry and word count (LIWC), neural embedding-based topic modeling, and time series analysis were utilized to build time series feature sets with lagging one, three, seven, and 14 days. Correlation analysis was used to examine the impact of COVID-19 on OPHS behaviors across different OMHS platforms. Machine learning algorithms and Shapley additive explanation (SHAP) were used to build the prediction. The result showed that the massive growth of OPHS behavior during the COVID-19 pandemic was a common phenomenon. The predictive model based on random forest (RF) and feature sets containing temporal features of the OPHS number, mental health topics, LIWC, and COVID-19 cases achieved the best performance. Temporal features of the OPHS number showed the biggest positive and negative predictive power. The topic features had incremental effects on performance of the prediction across different lag days and were more suitable for OPHS prediction compared to the LIWC features. The interpretable model showed that the increase in the OPHS behaviors was impacted by the cumulative confirmed cases and cumulative deaths, while it was not sensitive in the new confirmed cases or new deaths. The present study was the first to predict the demand for OMHS using machine learning during the COVID-19 pandemic. This study suggests an interpretable machine learning method that can facilitate quick, early, and interpretable prediction of the OPHS behavior and to support the operational decision-making; it also demonstrated the power of utilizing the OMHS platforms as an always-on data source to obtain a high-resolution timeline and real-time prediction of the psychological response of the online public.

## Introduction

Throughout the world, people are affected by mental health disorders at staggering rates (1). In many cases, people who lack appropriate treatment or have mental health conditions may experience severe human rights violations, discrimination, and stigma (2). COVID-19 has direct and indirect impacts on mental health conditions, while traditional mental health systems around the world are challenged during the pandemic, resulting in the disruption of their essential services. Online mental health service (OMHS) has been named as the best psychological assistance measure provided in the lockdown during the COVID-19 pandemic. The OMHS is conducive to saving time. More importantly, it has the advantage of avoiding face-to-face contact between patients and practitioners, which is critical to curb the spread of the COVID-19 successfully (3).

During the COVID-19 pandemic, previous studies found that the pooled prevalence of psychological stress, anxiety, depression, and posttraumatic stress symptoms among the general population were 29.6, 31.9, 33.7, and 23.9%, respectively, till the end of May 2020 (4). Similarly, high prevalence rates of acute stress, fear, anxiety, and depression symptoms were also observed in China (5). Compared to the prevalence rates of psychological diseases before the pandemic, the prevalence rates during the pandemic increased sharply among the general population in China (6). Therefore, it is reasonable to suspect that the demand for OMHS would increase during the COVID-19 pandemic due to the increased prevalence rates of psychological problems.

Considering the continuing influence of the COVID-19 pandemic on the mental status of the public, building an interpretable, accurate, and early prediction for the demand of OMHS is crucial for local governments and organizations which need to allocate and make decisions in mental health resources. Machine learning techniques have been widely applied in mental healthcare to facilitate the automatic detection of psychiatric diagnoses, such as suicide risks (7, 8), depression (9, 10), and to monitor system trends to predict the outbreak of psychological crisis (11, 12). Despite the successes, machine learning has its own limitations and drawbacks. The most significant one is the lack of transparency behind their behaviors (13), which leaves users with little understanding of how particular decisions are made by these models.

The interpretability gives machine learning the ability to explain or to present their behaviors in understandable terms to humans (14), which would be an effective tool to mitigate these problems in the prediction of OPHS. From the perspective of taking immediate crisis response, using the machine learning method may provide more accurate predicted values of OPHS behavior, which enables governments and OMHS platforms to rationally organize and allocate valuable counselors based on the help-seeking trends. From the perspective of psychological intervention, interpretable machine learning methods can identify the underlying risk factors of the OPHS [e.g., the surge in COVID-19 cases, the massive unemployment (15), and decreased access to mental health services (16) etc.], which offers policy suggestions for governments to undertake the follow-up of psychological intervention strategies.

In this study, we used the daily OPHS number as an indicator of public demand for the OMHS. Therefore, the question concerned in the present study was which variables (i.e., features) can be utilized to predict and explain the OPHS behavior of the public in the context of the COVID-19 pandemic in China. According to previous studies, COVID-19 cases, mental health topics related to the OPHS behavior, linguistic features, and temporal features were expected to correlate with the OPHS behavior in the context of the COVID-19 pandemic (17–19).

More specifically, the first type of variable is the COVID-19 cases, which include cumulative confirmed cases, cumulative deaths, new confirmed cases, and new deaths. Previous studies found that the COVID-19 cases would affect the investment and trust behavior, and the physical activity of the public (20–22). The OPHS behavior affected by the COVID-19 cases was also investigated among the public workers and college students (23, 24). However, how the OPHS behavior of the Chinese public was affected by the COVID-19 cases has not yet been understood. That is, no previous study has investigated the OPHS behavior affected by the number of COVID-19 cases from the perspective of the Chinese public with psychological problems.

The second type of variable considered in the present study is the mental health topics related to OPHS behavior. According to the five stages of grief proposed by Kuber-Ross, people who experience grief would go through a series of five emotions, which include denial, anger, bargaining, depression, and acceptance (25). Supported by this model, people may experience these emotions sequentially and have psychological problems associated with these emotions during different stages of the COVID-19 pandemic.

Moreover, linguistic features were also considered in the present study when predicting the OPHS number during the COVID-19 pandemic. Previous studies found that depressed and anxious people expressed themselves differently in the language (26). If the online psychological help-seekers would seek help due to different psychological problems during the different stages of the COVID-19 pandemic, their expression and texts would change accordingly, which indicates that the linguistic features may be important predictive variables for predicting the OPHS number.

Last but not the least, temporal features of the OPHS number are considered in the present study to predict the OPHS number. Time-series analysis techniques have been used in the prediction of COVID-19 cases. It is reasonable to believe that the temporal features of the OPHS number extracted by time series analysis would be a strong predictive variable for the OPHS number during the COVID-19 pandemic.

The purpose of the present study was to build a predictive model for the OPHS behavior, then identify and investigate the influences of the above factors, which must meet the following two requirements. First, motivated by the considerations of practical applications, this model must predict the OPHS number in a relatively long term (one or 2 weeks) rather than in a short term (e.g., the same day or the next day) (27). Second, the present model would integrate an innovative method to provide some possible explanations for the predictive performance and to investigate how the LIWC and the mental health topics expressed in the OPHS, the COVID-19 cases, and the temporal features of OPHS number, influence the model. Overall, the present study aimed to build an interpretable machine learning model that could predict the OPHS behavior in long lag days during the COVID-19 pandemic. Besides, the importance and influence of the predictive variables were investigated for interpreting the model.

## Materials and Methods

### Data Crawling

The first data source is one of the largest Chinese OMHS platforms, “One Psychology Community” (28), through which about 20 million people have asked for mental health services. People could anonymously post their psychological problems and seek psychological help and support from the psychological counselors in the platform of Q&A community. The question post could include the following optional components: the title of the question, age and gender of the help-seeker, course of the psychological problem, inner feelings, duration of the problem, and the label (i.e., occupation, marriage, romantic relationship, family, etc.). We utilized “Bazhuayu” (29), a web scraping software, to crawl 54,797 psychological help-seeking questions ranging from January 31, 2018, to January 08, 2021, of which 3,263 posts referred to the COVID-19 pandemic. The average daily OPHS numbers per day was 29.93. Each post contained three sections, i.e., the description of the title, the description of the psychological problem, and the asking time. The report conducted by a famous Chinese online counseling platform, “JianDanXinLi” (30) in 2020 showed that among the visitors of OMHS users, the female visitors were more, who were three times more than the male visitors, and visitors in the early adulthood (21–35 years old) accounted for 77.57%.

The second data source is the official website of the National Health Commission (31) through which search could be done on the COVID-19 cases in China including cumulative confirmed cases, cumulative deaths, new confirmed cases, and new deaths (32).

The third data source is the MOE-CCNU mental health service platform (the MOE-CCNU OMHS platform) (33), through which the time-series data on the daily OPHS number could be collected. Since January 31, 2020, the platform has been opened to psychological help-seekers *via* WeChat, which is the most popular social network app in China. Time-series data on the number of daily OPHS behaviors were collected from January 31, 2020, to January 08, 2021, with a total number of 37,698 OPHS behaviors.

### Data Analysis

#### Neural Embedding-Based Topic Modeling

Neural embedding is a family of techniques for obtaining a compact, dense, and continuous vector-space representations of entities that can efficiently encode multifaceted relationships among those entities (34), which has become a core ingredient in modern machine learning (35), and has recently offered novel opportunities and solutions to challenging problems, e.g., language evolution, gender, and stereotypes (36–39). In our study, for analyzing psycholinguistic clues (i.e., psychological problems and influential factors) in the OPHS behavior, we proposed a neural embedding method named, Word2vec (40) to learn dense and compact vector-space representations of mental health-related words in the OPHS question text.

Specifically, first, we constructed a predefined lexicon regarding the psychological problems and the influential factors of mental problems. Two Ph.D. candidates in Psychology extracted and categorized two types of seed words from sources that are directly related to mental health, e.g., Kessler 10 and Patient Health Questionnaire (41), the emotional vocabulary of Dalian Institute of Technology (42), and the question tag system of One Psychology (43).

Second, we constructed the domain lexicons of the OMHS community. We cut the texts of mental health questions and deleted stop words by using the Jieba tool (i.e., a Python segmentation package for Chinese) and the Baidu stop-word list. According to the word embedding algorithm, the texts were used as the training corpus. The word vector technology of Word2vecin Gensim software (44) was used to construct the vector model of mental health pretraining words for obtaining domain lexicons of psychological problems and related influential factors. Based on the word vector, we calculated the cosine similarity between the words in the vector model and the predefined vocabulary, to build the domain lexicons of psychological problems and influential factors. Specifically, the mental health lexicons contain two parts: (1) about 2,567 words related to the psychological problems of the OPHS. The semantic similarity between these words and the predefined seed words are >0.3260; (2) about 1,077 words related to the influencing factors of the OPHS. The semantic similarity between these words and the predefined seed words are >0.3556.

Third, we obtained the topics of the psychological problems and the influential factors of the help-seekers. We recruited two graduate students to set the cosine similarity thresholds to remove words in the OPHS texts which were irrelevant to the lexicons of the psychological problems or influential factors. The thresholds can improve the accuracy and interpretability of the topic detection, through which the formation of mutual interference between these two types of semantics could be avoided. Word vector representations of psychological problems and influential factors were obtained by using the average word embedding method (45). Based on these text vector representations, we used the k-means clustering algorithm (Python implement of K-Means method in scikit-learn) and its evaluation index (i.e., silhouette coefficient), to obtain and evaluate the clustering performance with different numbers of clustering centers (46). We tried 4–20 numbers of clustering centers. We finally selected the best k-mean clustering model with 7 cluster centers for the detection of topics. The number of clusters under the optimal silhouette coefficient was selected to construct the cluster of psychological problems and influential factors; refer to Supplementary Figure 1 for the details of models with different clusters and its silhouette coefficient. The values of the silhouette coefficient range from −1 to 1. A higher value represents a better clustering performance. Then, we recruited two Ph.D. candidates to classify similar topics of psychological problems and influential factors according to high-frequency keywords related to several clusters, and to determine the content and number of topics regarding the psychological problems and the influential factors of the help-seekers.

#### Time Series Analysis

Predicting the future trends is one of the most challenging but valuable tasks for scientists in the field of machine learning. We used the time series analysis method named, Prophet to identify the temporal features of OPHS behavior during the COVID-19 pandemic. Prophet is an advanced classical time series analysis method based on the generalized additive model developed by Facebook (47). It is capable of generating forecasts of a reasonable quality at scale. According to Taylor and Letham, Prophet always performs better than other classical approaches (47), and through which we can identify the trend of OPHS time series, such as yearly, weekly, etc. On this basis, we used the Pearson's correlation coefficient to quantify the relationships between daily OPHS numbers in OMHS platforms of MOE-CCNU and that in the OnePsychology during the COVID-19 pandemic.

#### Interpretable Machine Learning

We took the daily time series of the OPHS behavior in the Q&A section in the OMHS community as the dependent variable. We also took the frequency of the OPHS topics, the language clues in LIWC, the temporal features of the daily time series of the OPHS behavior, and the daily time series of the COVID-19 cases as independent variables. We utilized the regression method of machine learning to build the OPHS number predictive model with lagging one, three, seven, and 14 days and used the Shapley additive explanation (SHAP) method to investigate the predictive power of the features. The regression algorithms utilized in the present study included linear regression (LR), ridge regression (RR), least absolute shrinkage and selection operator (LASSO), support vector regression (SVR), and random forest (RF). The Prophet prediction method was used as a baseline of the classical time series prediction method. The result of 10-fold cross-validation related to RF is shown in Supplementary Table 1.

Interpretability is one of the key approaches in which the time series prediction method can be used to facilitate decision support. *Post-hoc* interpretable models are developed to interpret trained predictions, helping to identify important features or examples without modifying the original weights. Specifically, The SHAP method was considered as one of the two techniques for *post-hoc* interpretability in time series forecasting with machine or deep learning (48). The SHAP is a widely used approach based on the cooperative game theory, which comes with desirable properties. The SHAP represents responsibility of a feature for a change in the model output, which has at least two advantages (49). The first advantage of SHAP is the global predictability, i.e., it can show how much each variable contributes, either positively or negatively, to the target outcome. The second advantage is the local observability, i.e., each observation gets its SHAP value. Traditional machine learning interpretation only showed the results across the entire population but not in each case, while the local predictability of SHAP enables us to pinpoint and contrast the impacts of factors (13). The SHAP value greatly increases the transparency of machine learning and has been implemented in many studies and industry scenarios (48, 50).

Therefore, the RF regression and the SHAP value based on the interpretable machine learning framework were used to select the efficient features from the four predefined feature sets (i.e., topic, LIWC, temporal features of the OPHS number, and the COVID-19 cases; refer to Supplementary Table 2 for the details of these features). The mean absolute error (MAE) and Pearson correlation coefficient (Pearson Coef) was used to evaluate the performance of the predictive models. Then, we calculated the SHAP value for each feature and feature set in the best performance predictive model to investigate the ways through which the features contribute to the model.

We used accumulative SHAP values to quantify the positive and negative influence of the four feature sets on the OPHS number. If counting by days, the length of the time series is M. If the feature number of feature set F is {1, 2, …, *P*}, the SHAP values of the included features are


$$\begin{array}{l} \left[\begin{array}{ccc} SHAP_{1,1} & \cdots & SHAP_{1,P}\\ \vdots & \ddots & \vdots \\ SHAP_{M,1} & \cdots & SHAP_{M,P}\\ \; \end{array}\right], \end{array}$$

Therefore, the positive SHAP value of the feature set F is:


$$\begin{array}{l} SHAP_{F}^{+}n=\sum_{i=1}^{P}\frac{\left(\sum_{i,j=1}^{M}SHAP_{i}^{j}\right)}{X_{i}},SHAP_{i}^{j}>0,X_{i}\\ \;\in [X_{1},X_{2},\cdots ,X_{P}], \end{array}$$

*X*_*i*_ is the total number of positive *SHAP* values for an feature *i*. We calculated the positive SHAP value in the same way.

**Figure 1:**
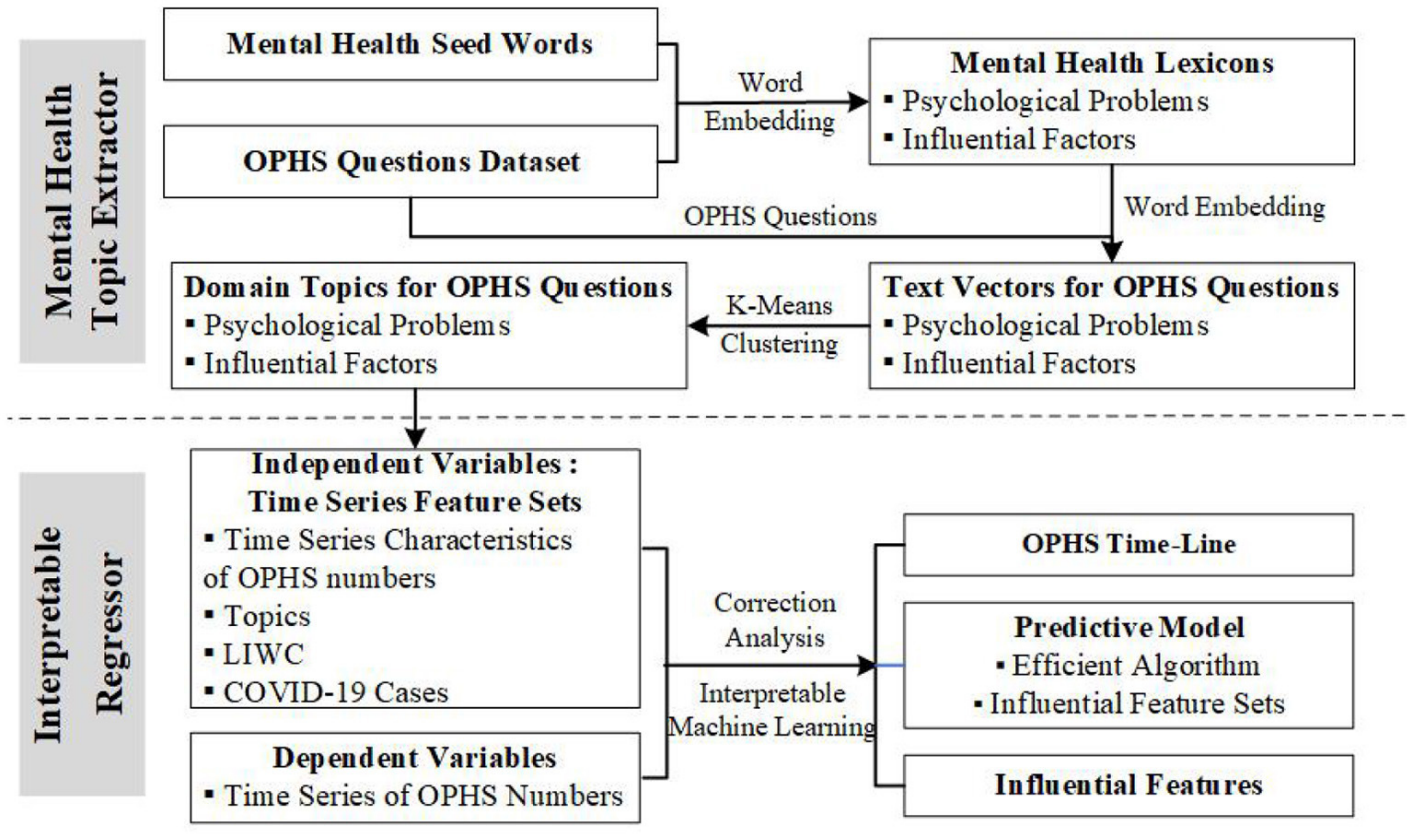
Research methods and processes.

The research methods and processes are shown in Figure 1. In summary, first, we obtained the OPHS behavior data of public by a web crawler named, “Bazhuayu,” mentioned earlier, from the OMHS community and the MOE-CCNU OMHS platform. Second, we used the existing knowledge related to psychological problems to construct domain lexicons by the neural embedding method. Then, we used the domain lexicons to remove words that were irrelevant to psychological problems in the OPHS texts, and obtained the vector representation of OPHS questions from every visitor, by neural embedding. We further used the k-means algorithm to cluster the vector representation of 'the OPHS questions of all visitors. The best clusters and related high-frequency words were validated manually. Third, we built the time series feature sets as the independent variables that contain the temporal features of the OPHS number, the COVID-19 cases, and mental health topics and LIWC features. We made the time series of the OPHS number as dependent variables. Finally, we built an interpretable machine learning model for predicting and interpreting the OPHS number, got the most effective algorithm and feature sets, and investigated the ways those features contributed to the performance of the predictive models.

## Results

### Analysis of the Timeline of OPHS Behavior and Related Psychological Problems and Influential Factors During the COVID-19 Pandemic

**Figure 2:**
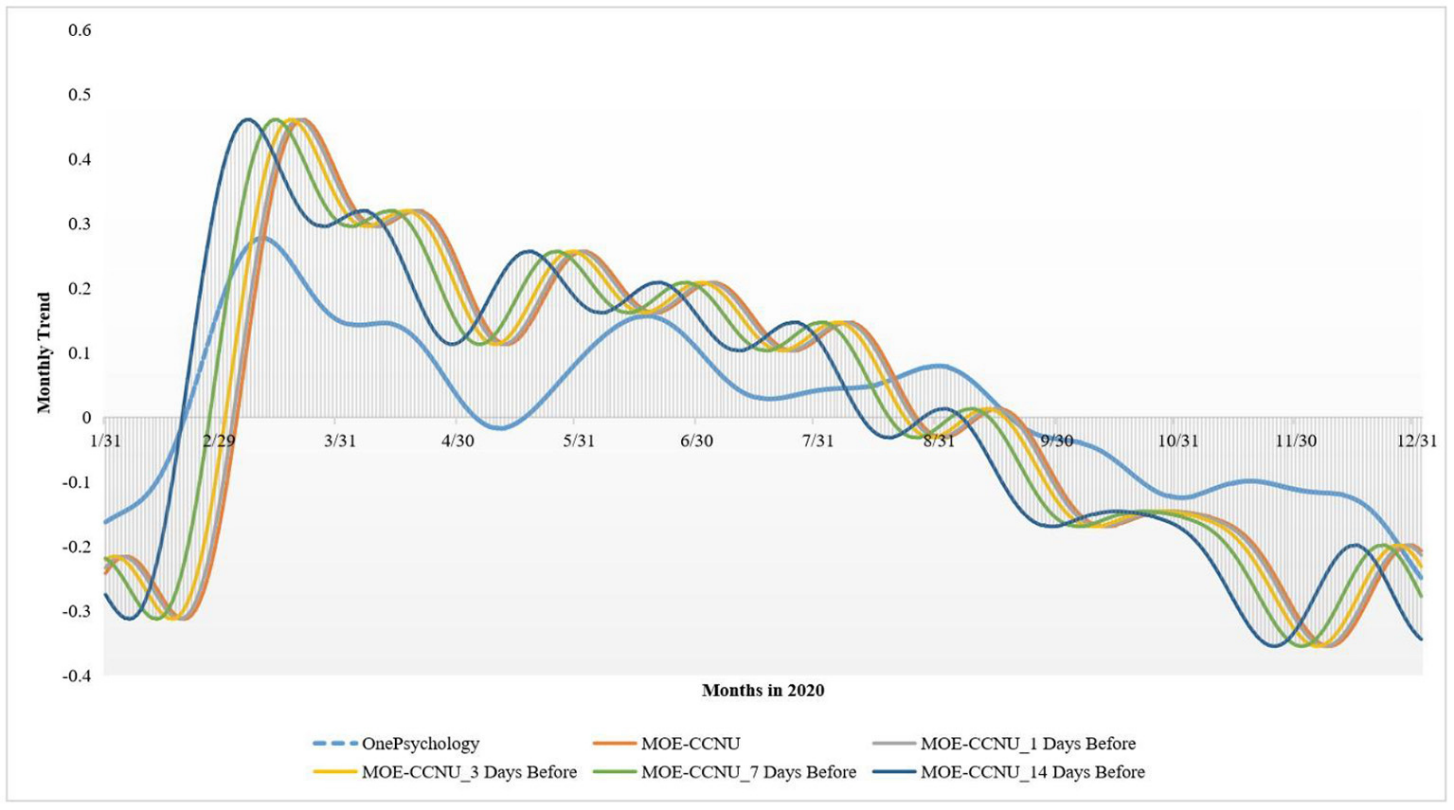
The trends of daily online psychological help-seeking (OPHS) numbers between online mental health service (OMHS) platforms of MOE-CCNU (MHSP) and the OnePsychology (OMHC) during the COVID-19 pandemic.

To validate the influence of the COVID-19 pandemic on the OPHS number, we utilized two OMHS platforms related to OPHS time-series data to recognize the trends of daily OPHS numbers in COVID-19. The OPHS trends of the two OMHS platforms with different lag days are shown in Figure 2. The result shows the OPHS behaviors in the OMHS community or the MOE-CCNU OMHS platform that increased sharply after the beginning of the COVID-19 pandemic. Specifically, compared to the OPHS behavior in the MOE-CCNU OMHS platform that peaked in mid-March, the OPHS behavior in the OMHS community peaked in early March. Further, as shown in Table 1, the correlation between the time series of the OPHS number in the OMHS community and platform was calculated. The OPHS number in the MOE-CCNU OMHS platform had the strongest correlation with that of the OMHS community with a lead time of 13 days, reaching 0.585 (*N* = 343, *p* < 0.05). The relationship between daily OPHS numbers in two OMHS platforms during the COVID-19 pandemic is shown in Table 1. The trends between the two daily OPHS numbers had a strong correlation as well, peaking at 0.911 with a lead time of 13 days (*N* = 343, *p* < 0.05).

**Table 1:** The correlations between the time series of the online psychological help-seeking (OPHS) number in the online mental health service (OMHS) community and platform.

**Lag days of OMHS platform**	**Correlations of time series of the OPHS number in OMHS platform and community**	**Correlations of the trends of the OPHS number in OMHS platform and community**
0	0.456^**^	0.794^**^
1	0.396^**^	0.811^**^
3	0.361^**^	0.841^**^
5	0.374^**^	0.868^**^
7	0.357^**^	0.889^**^
9	0.357^**^	0.904^**^
11	0.560^**^	0.911^**^
13	0.585^**^	0.911^**^
14	0.475^**^	0.908^**^

** *p < 0.001*.

By the topic modeling of the OPHS texts, we extracted seven psychological problems, seven influential factors, and the corresponding keywords (refer to Table 2). The topics of the psychological problems included depression and anxiety, suffering, social phobia, lack of interest, suicidal tendency, worry (afraid), and anger. The topics of influential factors involved love, marriage, psychotherapy, work, interpersonal relationship, personal characteristics, and family.

**Table 2:** The mental health topics related to the OPHS behavior.

**Type of topics**	**Topics**	**High-frequency word (ranking in order)**
Psychological problems	Depression and anxiety	depression, anxiety, insomnia, obsessive-compulsive disorder, depressive symptoms, diagnosis, bipolar, despair, violence, shadows, trauma, extreme, headaches, waking up, staying up, dreaming
	Suffering	unhappy, sad, uncomfortable, wronged, embarrassed
	Social phobia	communication, self-abasement, introversion, sensitivity, lack of self-confidence, dissocial, cowardice, dependence, eye contact, avoidance, conversation
	Lack of interest	no interest, no drive, no confidence, no enthusiasm, no desire
	Suicidal tendency	suicide, self-harm, tendency, breakdown, fear of pain, despair, escape, regret, torture, bad
	Worried, afraid	fear, worry, tension, doubt, struggle, avoidance, rejection, nausea
	Angry	anger, dislike, tantrums, bullying, grievance, disgust, blame, rejection, excess, ugliness, grumpiness, dissatisfaction, selfishness, trust, respect
Influential factors	Love	love, boyfriend, relationship, girlfriend, heterosexual, confession, break up, good feeling, gay, single, Ex, meet, ex-boyfriend, reunion, first love, ex-girlfriend, Cold War, entanglement, long-distance relationship
	Marriage	marriage, divorce, children, pregnancy, wife, man, mother-in-law, husband, married, sex, birth, in-laws
	Psychotherapy	treatment, diagnosis, pandemic, anxiety, disorder, medication, mental illness, withdrawal, bipolar, character, cognition, character disorder, schizophrenia
	Work	job, graduation, resignation, income, economy, pressure, development, unemployment, job-hopping, career, boss
	Interpersonal relationship	communication, character, contact, friend, speech, relationship, conversation, eye contact, dealing, indifference, impression, avoidance
	Personal characteristics	character, emotion, life, growth, cognition, conflict, obstacle, age, communication, impression, shadow, avoidance, dominance, character disorder
	Family	parents, mother, family, mom, father, dad, brother, grandmother, sister, daughter, grandparents

### Predictive Model for the Daily OPHS Number

For predicting the OPHS number in different lag days and investigating the importance of different features and feature sets, we tried to get a regression model with the best performance based on the refined feature sets.

As shown in Table 3, the RF achieved the best performance when lagging 3 days, and the ratio of MAE to the average OPHS number was 20.03% (5.99/29.93^*^100%). The SVR (a linear kernel function) achieved the best performance when lagging 1 day, 7 days, and 14 days. The ratios of MAE to the average OPHS number were 20.11, 21.14, and 22.84%, respectively. Overall, the RF and SVR performed better than other typical regression algorithms.

**Table 3:** Mean predictive performance of different algorithms for the OPHS number.

**Algorithms/** **lag days (day)/** **MAE**	**1**	**3**	**7**	**14**
LR	25.945	36.300	33.814	24.655
Ridge	6.440	6.717	6.781	7.014
LASSO	8.307	8.453	8.763	9.491
SVR	6.018	6.152	6.328	6.836
RF	6.280	5.995	6.398	7.790

*The average daily OPHS number is 29.929*.

Then, we compared the performances of different combinations of the four feature sets based on the RF regressor. As shown in Table 4, as for the performance of the single feature set in the prediction, the temporal features of the OPHS number performed better than the others. Notably, as lag days increased, the performance of the single feature set decreased. The combination of all the four feature sets showed a better performance than any single feature set with any lag days. However, the combination of four feature sets did not show the best performance at all the time, e.g., although the combination of the four feature sets achieved the best performance when lagging 14 days, it did not perform better than the combination of topic, time series, and COVID-19 cases when lagging 1 day, 3 days, and 7 days. Moreover, compared to the advanced time series forecasting method named, Prophet, the predictive model with the four feature sets achieved a better performance when lagging 3 and 7 days. In addition, there are similarities in the results between the correlation coefficient and the MAE. We can see that the prediction with a long lead time has a high correlation between its predicted and true values, although their MAEs are high.

**Table 4:** Predictive performance of the combinations of feature sets.

**LeadTime (day)/** **feature sets**	**Metrics**	**LIWC**	**Topic**	**Temporal features of the OPHS**	**COVID-19 cases**	**The combination of all four feature sets**	**Optimal features combination**	**Performance of the optimal combination**	**Prophet**
1	MAE	7.758	7.392	6.550	11.882	6.265	Topic & Timeseries & Covid19 pandemic	6.211	5.93
	Pearson Coef	0.731^**^	0.781^**^	0.798^**^	0.822^**^	0.884^**^		0.898^**^	0.923^**^
3	MAE	8.149	7.705	6.214	13.396	5.929	Topic & timeseries	5.780	5.96
	Pearson Coef	0.876^**^	0.898^**^	0.885^**^	0.906^**^	0.911^**^		0.932^**^	0.911^**^
7	MAE	8.258	8.343	6.470	11.947	6.400	Topic & timeseries	6.223	6.34
	Pearson Coef	0.916^**^	0.876^**^	0.913^**^	0.913^**^	0.924^**^		0.940^**^	0.908^**^
14	MAE	9.258	8.881	8.416	12.953	7.779	LIWC & topic & Timeseries & Covid-19 pandemic	7.779	5.92
	Pearson Coef	0.928^**^	0.93^**^	0.931^**^	0.901^**^	0.942^**^		0.942^**^	0.903^**^

** *p < 0.001*.

### Influential Factors of the Psychological Help-Seeking Behavior

To investigate the influence of the feature sets on the OPHS number, we calculated the cumulative SHAP values for different feature sets, as shown in Table 5. The result shows that the temporal feature set of the OPHS number is the largest positive and negative predictive power. The predictive power of LIWC was larger than that of the overall topic. The predictive power of the COVID-19 cases was larger than that of the topics but smaller than that of LIWC, but its positive and negative predictive power was stronger than both LIWC and topic feature sets when lagging 14 days.

**Table 5:** The impact of different feature sets on the OPHS behavior with different lag days.

**FeatureTypes/** **SHAP values/** **lag days(day)**	**Cumulative SHAP values (positive)**				**Cumulative SHAP values (negative)**				**Sum of absolute value of both – and +**
	**1**	**3**	**7**	**14**	**1**	**3**	**7**	**14**	
LIWC	10.998	7.344	9.093	4.768	−4.502	−3.300	−4.476	−3.010	47.490
Topic	6.306	8.045	1.615	3.199	−0.865	−1.003	−1.158	−0.929	23.120
Temporal features of the OPHS	48.097	56.710	52.345	48.286	−7.960	−15.160	−17.096	−9.848	255.503
Covid19_Pandemic	8.810	3.726	2.534	10.942	−3.834	−0.536	−1.422	−5.154	36.956

**Figure 3:**
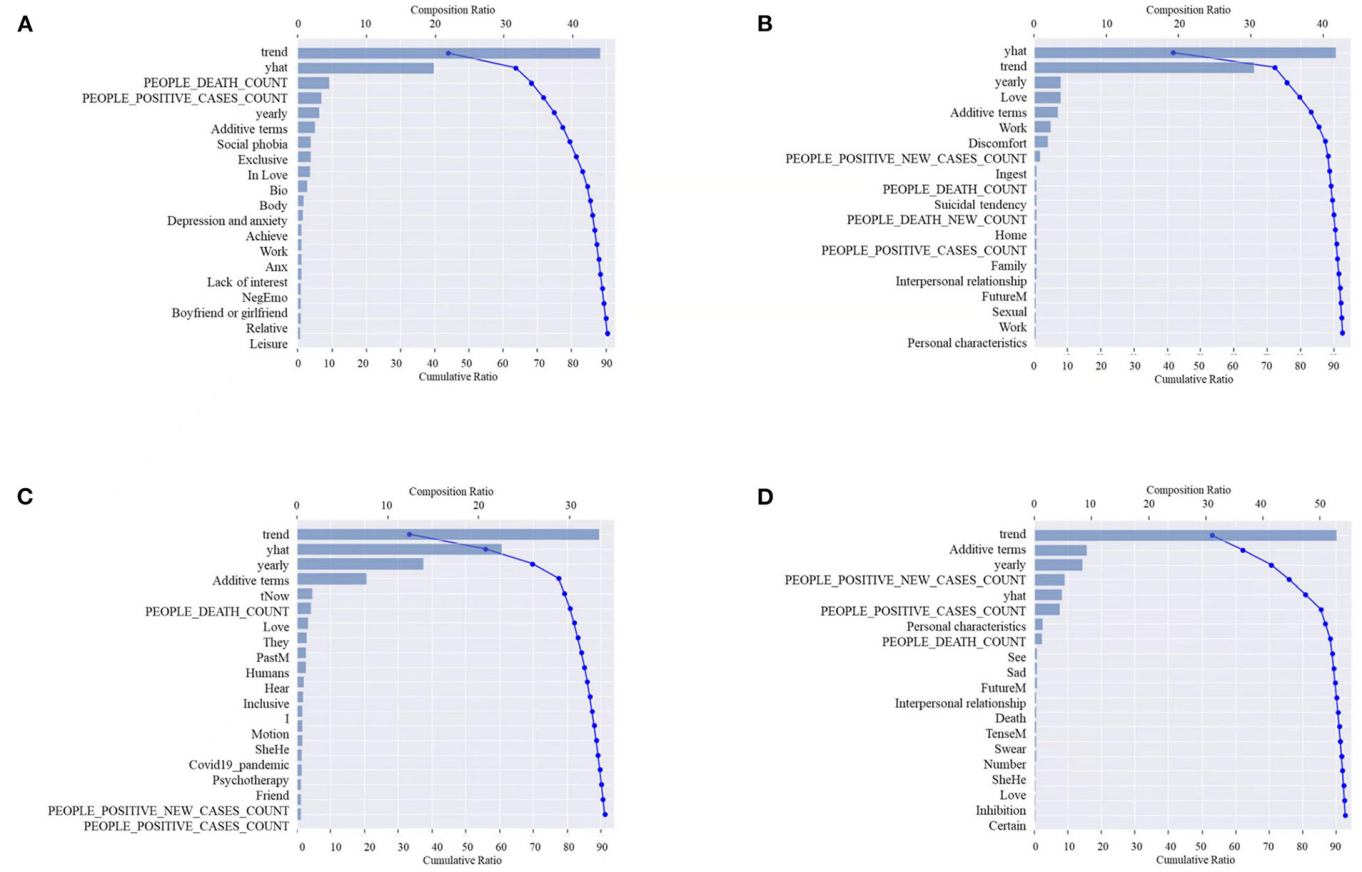
Top-20 features of predictions with lagging one **(A)**, three **(B)**, seven **(C)**, and 14 days **(D)**.

To quantify the cumulative contribution of different features in different predictions, we calculated the cumulative SHAP values of the top-20 features in the predictive model with lag days of 1, 3, 7, and 14 days, as shown in Figure 3. The top-20 features contributed more than 90% to the prediction with any lag days. The top-7 features contributed ~80% to the prediction with any lag days. Table 6 shows the top-20 features in predictions with different lag days. Among these features, temporal features of In OPHS numbers (i.e., trend, additive terms, year, yhat; Refer to Supplementary Table 2 for details, the same below.), COVID-19 cases-related features (i.e., people positive cases count and people death count) were included in the top-20 features of all the four models with different lag days. The LIWC feature (i.e., love) was included in the top-20 features in all the models with all four lag days except for 1 day. Other top-20 features in different lag days included some features in the LIWC features, e.g., personal pronouns (i.e., I, She, He, and They), number, informal language (i.e., swear), time orientations (i.e., TenseM, FutureM), social processes (i.e., friend and humans), Affective processes (i.e., NegEmo, Anx, and Sad), cognitive processes (i.e., certain, inhibition, inclusive, and exclusive), perceptual processes (i.e., see, hear, and bio), biological processes (i.e., body, sexual, and ingest), relative processes (i.e., relative and motion), personal concerns (i.e., work), drives (i.e., achieve), personal concerns (i.e., leisure, home, death, and love), and time orientations (i.e., tPast and tNow). Some features in the psychological problems and influential factors in the mental health topics are also the top-20 features in a model of the specific lag days, e.g., depression and anxiety, suffering, social phobia, lack of interest, suicidal tendency, love, work, social interaction, personal characteristic, and family.

**Table 6:** Top-20 features in predictions with different lag days.

**Top rank features**	**Lag days (day)**			
	**1 day**	**3 days**	**7 days**	**14 days**
Top 1	trend	yhat	trend	trend
Top 2	yhat	trend	yhat	Additive terms
Top 3	PEOPLE_DEATH_COUNT	yearly	yearly	yearly
Top 4	PEOPLE_POSITIVE_CASES _COUNT	Love	Additive terms	PEOPLE_POSITIVE_NEW_ CASES_COUNT
Top 5	yearly	Additive terms	tNow	yhat
Top 6	Additive terms	Work	PEOPLE_DEATH_COUNT	PEOPLE_POSITIVE_CASES _COUNT
Top 7	Social phobia	Suffering	Love	Personal characteristics
Top 8	Exclusive	PEOPLE_POSITIVE_NEW _CASES_COUNT	They	PEOPLE_DEATH_COUNT
Top 9	In Love	Ingest	PastM	See
Top 10	Bio	PEOPLE_DEATH_COUNT	Humans	Sad
Top 11	Body	Suicidal tendency	Hear	FutureM
Top 12	Depression and anxiety	PEOPLE_DEATH_NEW_COUNT	Inclusive	Interpersonal relationship
Top 13	Achieve	Home	I	Death
Top 14	Work	PEOPLE_POSITIVE_CASES _COUNT	Motion	TenseM
Top 15	Anx	Family	SheHe	Swear
Top 16	Lack of interest	Interpersonal relationship	covid19_pandemic	Number
Top 17	NegEmo	FutureM	Psychotherapy	SheHe
Top 18	Boyfriend or girlfriend	Sexual	Friend	Love
Top 19	Relative	Work	PEOPLE_POSITIVE_NEW _CASES_COUNT	Inhibition
Top 20	Leisure	Personal characteristics	PEOPLE_POSITIVE_CASES _COUNT	Certain

**Figure 4:**
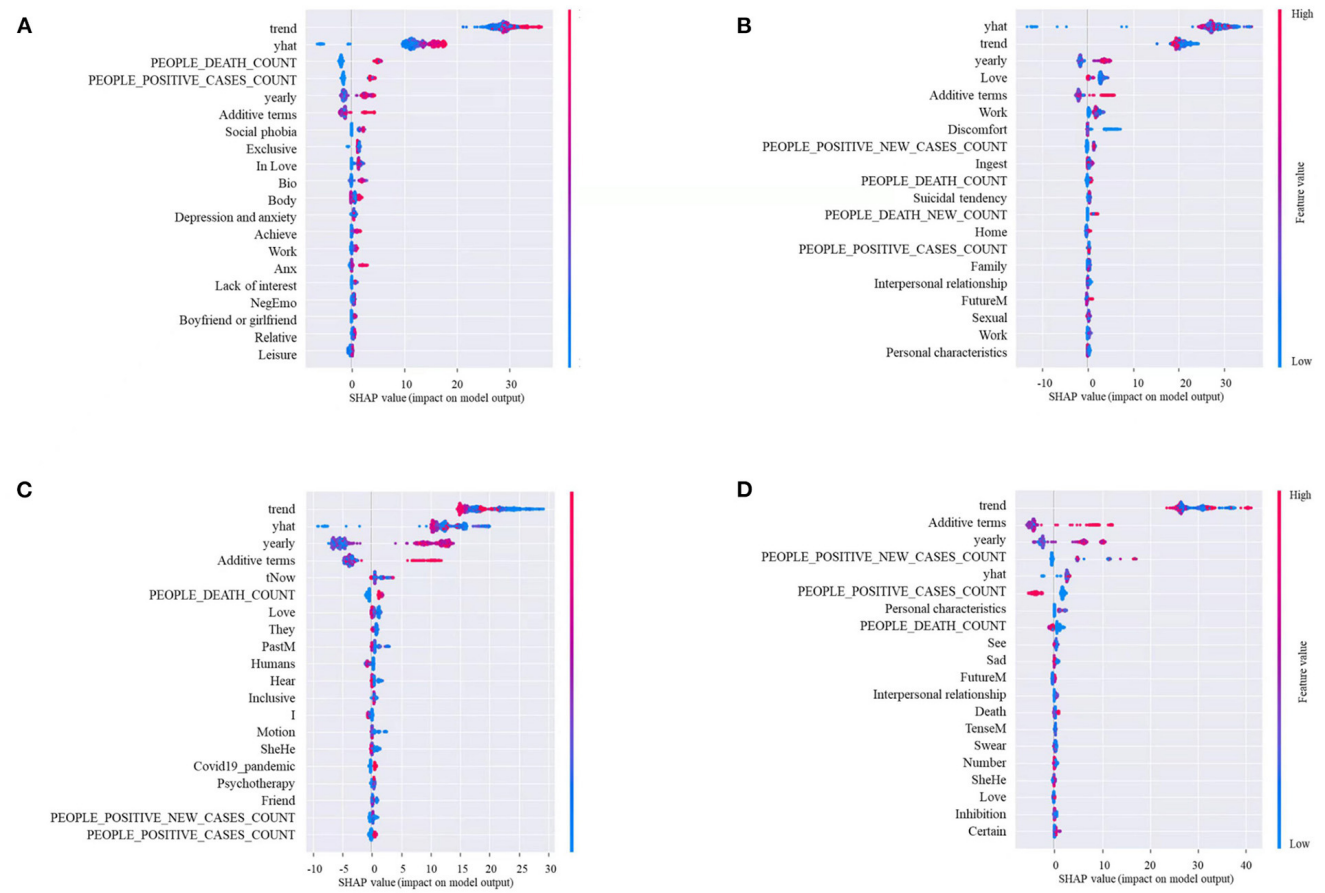
The Shapley additive explanation (SHAP) summary plots about the adjustment to the predicted In OPHS numbers (x-axis) for each of the top-20 features with lagging one **(A)**, three **(B)**, seven **(C)**, and fourteen days **(D)**.

To understand how the ways features contribute to the performance of the predictions, we summarized the influential ways of top-20 features on the OPHS number, as shown in Figure 4. The figure shows the adjustment to the predicted x-axis for each of the top-20 features. Each plot is made up of thousands of individual points from the predictive dataset. As the higher value is redder, the lower value is bluer. This is depicted by the feature value bar on the right of each plot. Besides, if the dots on one side of the central line are increasingly red or blue, it suggests the increasing values or declining values, prospectively. For instance, lower “Trend” values (blue dots) are associated with a relatively lower OPHS number.

The result showed that the temporal features of daily OPHS numbers (i.e., trend and yhat) positively predicted the OPHS number in all lag days. The LIWC features (i.e., love) positively predicted the OPHS number when this feature was at a lower level, while negatively predicted the number when it was at a higher level with lagging 3, 7, and 14 days. The additive terms, the yearly trend in the temporal features of the OPHS number, the COVID-19 cases (i.e., people positive cases count and people death count), the number, biological processes (i.e., body and ingest), time orientations (i.e., tNow), personal concerns (i.e., death), cognitive processes (i.e., certain), perceptual processes (hear), relative processes (motion), time orientations (i.e., FutureM), social processes (i.e., Humans), affective processes (i.e., Anx, NegEmo), perceptual processes (i.e., Bio) in the LIWC features, and suffering, depression and anxiety; social phobia in the topic features positively predicted the OPHS number when these features were at high levels, while negatively predicted the number when they were at low levels.

## Discussion

### Principal Results

The present study built four types of feature sets (i.e., LIWC, mental health topics, temporal features of the OPHS number, and the COVID-19 cases), and used the machine learning method (i.e., LR, RR, LASSO, SVR, and RF) to predict and interpret the daily OPHS number during the COVID-19 pandemic. We found several interesting findings as follows.

First, after the beginning of the COVID-19 pandemic, the daily OPHS number in both the OMHS community and the MOE-CCNU OMHS platform increased significantly, and the number of help-seekers in the OMHS community reached the peak at 13 days earlier than that in the OMHS platform. Moreover, a strong and positive relationship between daily OPHS numbers in the OMHS platforms of MOE-CCNU and that in the OnePsychology, indicated that the dynamic changes of the OPHS behavior of the online public was not an exception.

Second, for the performance of predictions with different feature sets, we found that the model with feature sets containing temporal features of the OPHS number, mental health topics, LIWC, and COVID-19 cases under RF or SVR regression achieved the best performance. (1) Although the feature set containing all the four types of features performed overall better than any single feature set, it cannot always perform the best. For example, when predicting the OPHS number with a lagging of 14 days, the best performance was obtained by using all four types of features. Nevertheless, when predicting the OPHS number with lagging 3 or 7 days, the best performance was obtained by using only two types of features (i.e., topic and temporal features). This finding can be supported by the principle of feature selection, i.e., more features do not necessarily lead to better performance because of redundancy and the irrelevance of features (51). (2) The present study found that the temporal features of the OPHS number have an advantage over other features in the prediction. For example, the models with lagging 1, 3, and 14 days show that the trend of daily OPHS numbers might be the most important feature, followed by predicting values and yearly trend generated by the Prophet. A possible explanation is that the temporal features contain more information, such as the cyclical and trend changes affected by the environment and events (47). (3) Compared to LIWC, the topic features we proposed were more important and had incremental effects on the overall performance of models with different lag days, which indicated that mental health-related linguistic features were more targeted to OPHS behavior prediction. It could be supported by a previous study which found that the LIWC model performs better in the document with approximately 22 sentences while the topic model performs better in the document with about two sentences (52). The help-seeking posts are usually short and express their psychological problems, which implies that the topic model performs reasonably better.

Third, for the performance of predictions with different lag days, our models were predictive for the number of OPHS with lag days up to 2 weeks. Compared to an advanced classical forecasting method named, Prophet, the present model has advantages when lagging 3 and 7 days and has interpretability that the Prophet does not have. The present predictive model may help to facilitate early, fast, and accurate prediction and interpretation for the daily OPHS number in the context of a major public health emergency. Meanwhile, it can help the government and platform managers to arrange the number of psychological consultants on duty reasonably, and to take targeted interventions and public policy to prevent potential psychological crises of the online public.

In particular, with respect to the explanation of the model built in the present study, we found some meaningful results.

First, we found that the top-20 features included trend, additive terms, yearly, yhat in temporal features of the OPHS number, people positive cases count, and people death count in COVID-19 cases among all the four models, which indicated that these features might be the most important ones for predicting the OPHS number.

Second, the results from the SHAP values provided possible explanations for the black-box models, which broke the stereotype that machine learning methods were difficult to interpret and understand. It is crucial to gain a better understanding of the ways the features contribute to the performance of the predictive model. For example, in the cumulative confirmed cases, the cumulative deaths positively predicted the OPHS number when these features were at high levels, while negatively predicted the OPHS number when they were at low levels, which indicated that the increase in the OPHS number was affected by the cumulative confirmed cases and cumulative deaths, while it was not sensitive to the new confirmed cases or new deaths. The effect sizes of these two COVID-19-related features got larger when predicting the OPHS number with longer lag days. Considering the individual mental health status that changes continuously, sporadic new confirmed cases or new deaths of the COVID-19 may not have a great impact on the OPHS behavior of the public. However, the impact of major changes in the social environment on the mental health of the public is profound and lasting (53). The present study indicates that this phenomenon is also reflected in the growth of OPHS behavior. Therefore, governments and institutions should continue to support online mental health services, focus on top-ranked problems of online psychological help-seekers with regard to depression and anxiety, suffering, social phobia, lack of interest, suicidal tendency, worried and afraid, and anger, then cultivate online psychological assistance force related to these problems and take targeted interventions for online psychological help-seekers at different stages of the COVID-19 pandemic.

Third, other influential factors which had small or medium effect sizes are also worthy of attention. (1) The results indicate that linguistic clues of biological processes related to body and interest are relevant to the increase of OPHS behavior of the public. This is consistent with the previous studies that proposed that chronic diseases lead to poor mental health (54). Therefore, OMHS may be an option for hospitals to deal with mental diseases related to traditional physical diseases during the COVID-19 pandemic. (2) The results indicate that the increase in the linguistic clues of perceptual processes related to hearing, and cognitive processes related to certainty are related to the increase in the OPHS behavior of the public. Previous studies have pointed out that mental health problems are accompanied with abnormal states of individual perception and cognition (55). These abnormal problems may be related to the increase in the OPHS behavior of the public. (3) The results show that the linguistic clues of the topics related to social processes and social phobia are related to the growth of the OPHS behavior of the public. For example, previous research on teenagers found that individuals with a stronger connection to school are less likely to have mental health problems, such as depression and anxiety (56). The present study found that the problematic connection between individuals and the social environment are related to the increase in the OPHS behavior of the public. (4) The results show that linguistic clues of the affective processes related to anxiety and negative emotion, as well as the topics related to suffering, depression, and anxiety are related to the increase in OPHS behavior. Previous studies found that negative emotions significantly affect individual mental health and lead to depression (57). The present study found that these emotional problems are related to the growth of the OPHS behavior of the public. (5) The result shows that the linguistic clues of personal concern related to death are related to the OPHS behavior of the public. As suicidal tendency related to greater help-seeking and perceived need (58), the positive relationship between deaths and the OPHS behavior is supported.

### Strengths and Limitations

The present study has some strengths and limitations which need to be considered when weighing the findings. The following strengths are found in the present study.

To the best of our knowledge, the present study was the first to predict the OPHS behavior using the machine learning method in China in the context of the COVID-19. We considered four types of features, which avoided the underfitting problem caused by a single type of feature. This research seems to be a competitive illustration of the power of always-on mental health data sources: if we had used traditional data sources, we would not have obtained such a high-resolution timeline and real-time prediction of the immediate mental health response of the public to an unexpected event, such as the COVID-19 pandemic.

Specifically, first, despite the successes of machine learning in mental healthcare, the concerns about the black-box nature of these complex models have hampered their further applications, especially in those critical decision-making domains like policy responses to COVID-19. The present study proposes an interpretable machine learning method that makes the predictions easy to understand and supports operational decision-making. This could help governments and organizations identify risk factors for the increase in OPHS behavior. For example, unemployment has been proven to be an influential factor in the increase in the OPHS number related to psychological crisis during the COVID-19 pandemic (15), as the factor of unemployment was included in the present predictive model. Thus, by analyzing the discourses of the OPHS, the prediction can quickly sense the emergencies and the changes of risky, predictive factors, and help governments and organizations in making policy tools and administrative interventions for the public mental health.

Second, previous prediction studies driven by big data from social media tended to believe that measurement in big data sources was much less likely to change behavior, namely the nonreactivity. However, even though some big data sources are nonreactive, they are not always free of social desirability bias, as people always want to present themselves in the best possible way (59). For example, as one respondent in an interview-based study said, “It's not that I don't have problems, I'm just not putting them on Facebook” (60). Therefore, nonreactivity does not ensure that these data can direct reflect psychological problems of people to some degree in social media-based mental health prediction of the public. The present study used the always-on anonymous OPHS data, enabled the investigation of unexpected mental health events, and real-time measurement for the status of public mental health.

Third, previous studies tracked mental status of people on a large scale in social media including Facebook and Twitter without obtaining their consent and awareness have raised ethical concerns (61). The present study found that the continuous operation of the anonymous online mental health community in big data systems could enable researchers to study emergencies and provide real-time information for decision-makers, while could also avoid this problem.

The present study is not without limitations. First, the large sample size in the present study limited the possibility of selection bias. However, we have to admit that the topic features need to be further explored. Although the topic features perform better than the LIWC features when they are used alone, the topic features in the best predictive model did not play a relatively important role, which was inconsistent with our hypothesis. One possible explanation is that the topic features (14 dimensions) contain much fewer dimensions compared to the LIWC (101 dimensions). Therefore, topic features not only have competitive positive and negative predictive power compared to LIWC, but the dimensions in the topics also have stronger average predictive power, so its prediction of the OPHS number is more targeted. Another possible explanation is that the OPHS number changed for each type of psychological problem, while the overall number of the OPHS behavior remained stable.

Lastly, compared to the classical time series forecast method, the proposed method does not achieve absolute advantages on all lag days. The reason may be that we can only get relatively few OPHS data when conducting this study (in 2020 during the COVID-19 pandemic). Subsequent research can collect more data and use deep learning forecasting methods to improve the existing results.

### Implications

A previous study points out that there is an explosive increase in OPHS after the outbreak of the COVID-19, and the OPHS number varies across different stages during the pandemic (62). From the perspective of taking immediate crisis response, the use of machine learning techniques may provide more accurate predicted values of OPHS behavior, which enables governments and OMHS platforms to rationally organize and allocate valuable counselors based on the help-seeking trends.

**Figure 5:**
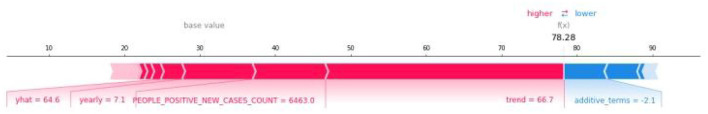
The SHAP force plots for a number of the OPHS prediction. The number of psychological help-seeking (PHS) rated in this example shows a prediction of 78.28 on the rating scale. In particular, the positive new case count of the people, equal to 6,463, increases its rating.

From the perspective of psychological intervention, using the interpretable machine learning, we can explore the underlying risk factors (e.g., work, marriage, interpersonal relationship, etc.) that cause an increase in the OPHS behavior, which offers policy suggestions for governments to undertake follow-up psychological intervention strategies. Take the prediction of the peak (02/28/2020) of the OPHS number as an example. The local interpretability of this method predicted and explained the peak 14 days in advance (78.28 people), as shown in Figure 5. We can see that the new confirmed cases were 6,463, which ranked second among the influential factors. These findings allow governments and organizations identifying risk factors during the increase in the OPHS number in advance, such as the surge in COVID-19 cases, the massive unemployment, and decreased access to mental health services, to facilitate the use of targeted administrative measures.

The global interpretability of this method helps government, OMHS platforms, and researchers understand how risky factors influence the dynamics of psychological response of the public and contribute to the development of psychological interventions policy. For example, the significant growth in the risky factors of COVID-19 cases, and topics of work, money in the prediction may indicate that financial relief should be provided for the unemployed during the social isolation, and targeted psychological support should be delivered to the public who return to work and school. Propaganda about the pandemic should avoid misinformation and massive panic.

## Conclusion

The present study investigated and predicted the OPHS number in China during the COVID-19 pandemic. Predicting and interpreting the OPHS behavior has a greater practical significance. Rational arrangements of the number of psychological counselors in advance are very important, which not only avoid the waste of the human resources but also enable help-seekers to get help promptly, especially in China where the number of psychological counselors is limited.

By understanding the risk and the protective factors in the OPHS behavior, the government can take administrative measures to prevent the potential psychological crisis. Besides, the OPHS behavior reflects, on one hand, the mental health literacy of the public, and on the other hand the number of psychological problems among the public. Therefore, using the ecological paradigm and big data techniques to study help-seeking behavior is a valuable research field.

## Data Availability Statement

The data analyzed in this study is subject to the following licenses/restrictions: Raw data were generated at https://www.xinli001.com/. Derived data supporting the findings of this study are available from the corresponding author Yinghui Huang on request. Requests to access these datasets should be directed to yhhuang@ccnu.edu.cn.

## Author Contributions

YH, HL, and LZ conceptualized the study, were involved in writing, and original draft preparation. YH and HL conceived the methodology and performed a formal analysis. HL, YH, SL, ZZ, ZR, and WW were involved in the process of writing, reviewing, and editing. HL was involved in visualization. WW and YH were involved in the process of obtaining funding and acquisition. All authors have read and agreed to the published version of the manuscript.

## Funding

This research was funded by the National Natural Science Foundation of China: Research on adolescent internet adaptation-oriented optimization method for personalized information service (No. 71974072) and supported by the Collaborative Innovation Center for Informatization and Balanced Development of K-12 Education by MOE and Hubei Province (Grant number xtzd2021-013).

## Conflict of Interest

The authors declare that the research was conducted in the absence of any commercial or financial relationships that could be construed as a potential conflict of interest.

## Publisher's Note

All claims expressed in this article are solely those of the authors and do not necessarily represent those of their affiliated organizations, or those of the publisher, the editors and the reviewers. Any product that may be evaluated in this article, or claim that may be made by its manufacturer, is not guaranteed or endorsed by the publisher.

## Supplementary Material

Supplementary Figure 1 Silhouette Coefficient for K-Means model with different number of clusters.

Supplementary Table 1 Results of 10-fold cross-validation (Random Forest).

Supplementary Table 2 Details of features.
