# Supplementary figures and images for: Prediction of Online Psychological Help-Seeking Behavior During the COVID-19 Pandemic: An Interpretable Machine Learning Method
Source: Front Public Health. 2022 Mar 3;10:814366. doi: 10.3389/fpubh.2022.814366 (PMC8929708; doi:10.3389/fpubh.2022.814366)

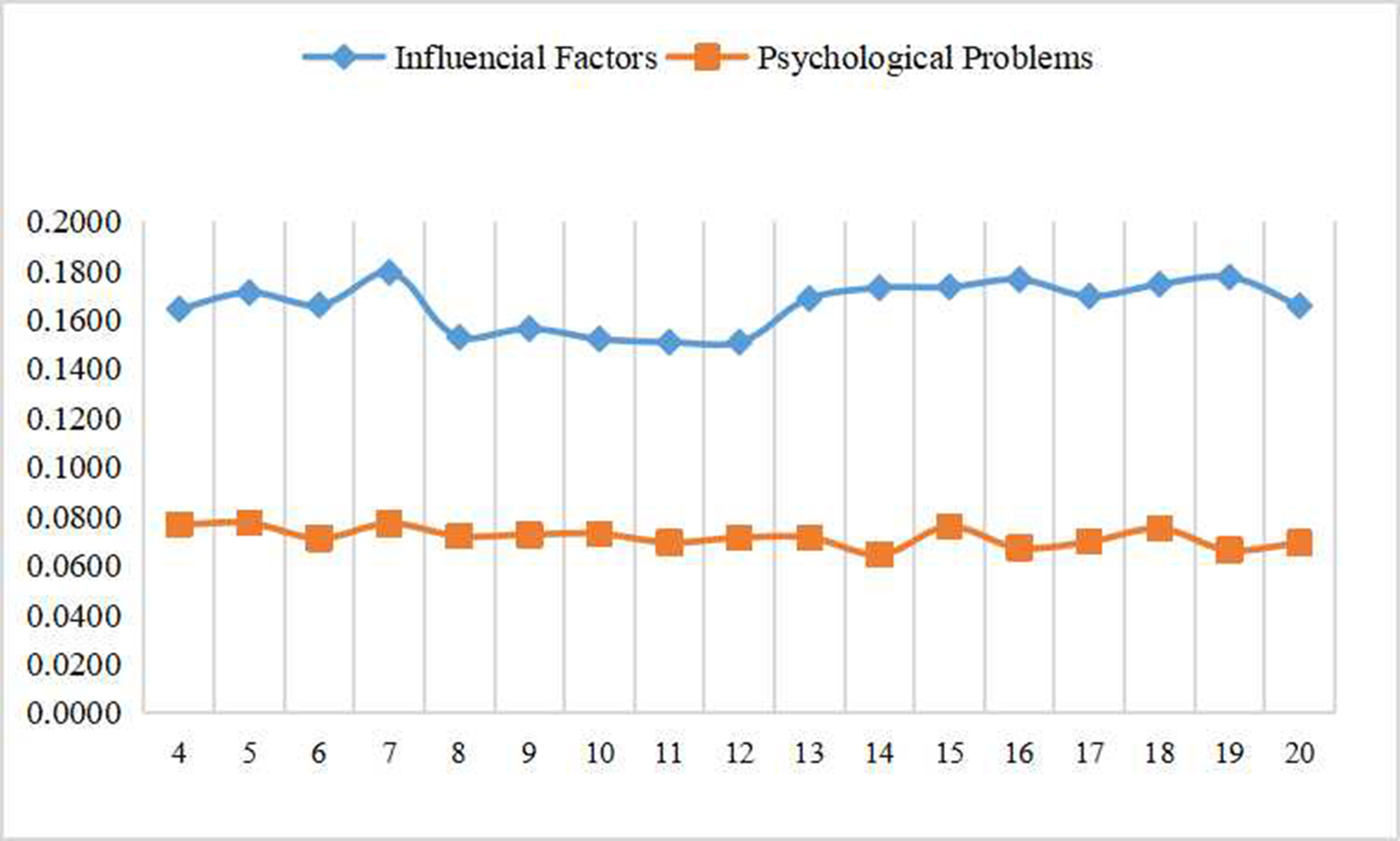

Supplement: Supplementary Figure 1 — Silhouette Coefficient for K-Means model with different number of clusters. [file Image_1.JPEG]
