# Supplementary material for: Prediction of Online Psychological Help-Seeking Behavior During the COVID-19 Pandemic: An Interpretable Machine Learning Method
Source: Front Public Health. 2022 Mar 3;10:814366. doi: 10.3389/fpubh.2022.814366 (PMC8929708; doi:10.3389/fpubh.2022.814366)
Supplement: Supplementary Table 1 — Results of 10-fold cross-validation (Random Forest). [file Data_Sheet_1.pdf]

Table A1 Results of 10-fold cross-validation (Random Forest)

**Leadtime(days)**	**Featuresets**	**Algorithms**	**Pearson Correlation Coefficient**	**1**	**2**	**3**	**4**	**5**	**6**	**7**	**8**	**9**	**10**	**Average**
**14**	LIWC	RandomForest	0.731**	10.12	9.54	4.76	5.04	12.22	29.36	20.76	8.69	21.41	13.14	14.45
**14**	Topic		0.781**	8.84	7.69	4.91	7.80	15.73	22.66	20.48	8.32	18.61	12.32	13.55
**14**	Timeseries		0.798**	12.04	10.80	5.76	4.30	11.43	18.17	20.96	8.19	16.48	11.94	13.13
**14**	Covid19_Pandemic		0.822**	10.03	9.45	4.73	5.07	12.28	23.23	12.44	11.64	10.54	10.88	11.97
**14**	LIWC&Topic&Timeseries&Covid19_Pandemic		0.884**	9.86	8.45	5.82	5.46	6.46	14.41	16.07	13.95	10.26	10.01	11.02
**7**	LIWC		0.876**	5.19	4.03	6.13	5.26	7.63	12.91	15.13	11.85	11.61	8.49	9.34
**7**	Topic		0.898**	5.53	6.05	5.19	3.69	6.15	9.53	18.09	7.76	12.16	7.98	8.77
**7**	Timeseries		0.885**	5.15	4.03	5.11	4.91	6.96	13.04	13.88	10.57	10.54	7.88	8.67
**7**	Covid19_Pandemic		0.906**	3.07	3.31	6.02	5.89	6.28	10.34	15.34	10.05	12.24	7.61	8.38
**7**	LIWC&Topic&Timeseries&Covid19_Pandemic		0.911**	4.52	5.62	5.31	3.89	5.34	13.61	16.22	7.74	9.21	7.51	8.26
**3**	LIWC		0.916**	3.89	5.13	6.14	3.74	5.33	11.52	17.65	8.55	7.76	7.37	8.11
**3**	Topic		0.876**	3.00	3.40	6.48	4.67	6.59	15.55	9.97	9.24	11.17	7.32	8.05
**3**	Timeseries		0.913**	3.22	3.23	6.15	5.31	5.88	9.81	13.38	10.40	10.43	7.14	7.86
**3**	Covid19_Pandemic		0.913**	3.09	3.11	4.93	5.01	6.60	7.69	15.31	9.21	11.23	6.94	7.64
**3**	LIWC&Topic&Timeseries&Covid19_Pandemic		0.924**	2.40	3.20	8.82	4.01	5.28	11.36	9.67	7.61	9.50	6.59	7.25
**1**	LIWC		0.928**	2.92	3.21	5.91	4.51	6.18	11.20	9.30	8.32	10.24	6.50	7.15
**1**	Topic		0.93**	2.43	3.28	7.31	3.71	5.15	10.03	12.52	8.05	7.82	6.38	7.02
**1**	Timeseries		0.931**	2.27	3.33	9.47	3.64	5.13	9.61	9.95	7.59	8.14	6.34	6.97
**1**	Covid19_Pandemic		0.931**	2.05	3.20	4.72	4.11	7.11	7.92	12.62	7.69	9.27	6.19	6.81
**1**	LIWC&Topic&Timeseries&Covid19_Pandemic		0.942**	2.54	2.66	4.76	4.31	5.61	5.97	12.45	7.69	8.90	5.76	6.34

Note: ** $p < 0.001$

Table A2 Table of Features' details

Feature Source	Name of the subclass to which the feature belongs	Name of the feature	Abbreviation of feature name	Example
LIWC Lingustic Clues	Affective processes	Emotional process words	Affect	Anger, gratitude, disappointment
LIWC Lingustic Clues		Positive emotion words	PosEmo	Confidence, satisfaction, blessing
LIWC Lingustic Clues		Negative emotion words	NegEmo	Worry, suspicion, revenge
LIWC Lingustic Clues		Anxiety words	Anx	Uneasiness, struggle, tension
LIWC Lingustic Clues		Angry words	Anger	Damn, complain, destroy
LIWC Lingustic Clues		Sad words	Sad	Heartache, depression, physics
LIWC Lingustic Clues	Social processes	Social process words	Social	Understanding, choosing, questioning
LIWC Lingustic Clues		Family words	Family	In laws, brothers, granddaughters

LIWC Linguistic Clues		Friend words	Friend	Companions, friends, comrades
LIWC Linguistic Clues	Cognitive processes	Insight words	Insight	Understand, suddenly realize, experience
LIWC Linguistic Clues		Causal word	Cause	Cause, let, become
LIWC Linguistic Clues		Gap Words	Discrep	Insufficient, expected, should
LIWC Linguistic Clues		Provisional words	Tentat	About, come on, almost
LIWC Linguistic Clues		Exact word	Certain	No doubt, necessity, guarantee
LIWC Linguistic Clues	Perceptual processes	Perceptual process words	Percept	Warmth, experience, gaze
LIWC Linguistic Clues		Visual words	See	Appearance, shiny, green
LIWC Linguistic Clues		Auditory words	Hear	Shout, hear, talk
LIWC Linguistic Clues		Sensory word	Feel	Smooth, elastic, touch

LIWC Linguistic Clues	Biological processes	Physiological process words	Bio	Dizziness, hugging, sweating
LIWC Linguistic Clues		Body words	Body	Neck, skin, stomach
LIWC Linguistic Clues		Health words	Health	Insomnia, doctor, exercise
LIWC Linguistic Clues		Sex words	Sexual	Sex, sex, nudity
LIWC Linguistic Clues		Feeding words	Ingest	Digest, eat and cook
LIWC Linguistic Clues	Drives	Achievement words	Achieve	Good at, responsible, expert
LIWC Linguistic Clues	Time orientations	Tense marker	TenseM	Already, before, in the future
LIWC Linguistic Clues	Relativity	Relative word	Relative	Before, compared to, reach
LIWC Linguistic Clues		Mobile word	Motion	Pass, participate, approach
LIWC Linguistic Clues		Spatial words	Space	Inside, street, stage

LIWC Linguistic Clues		Time word	Time	Period, past, autumn
LIWC Linguistic Clues	Personal concerns	Working words	Work	Factory, interview, salary
LIWC Linguistic Clues		Leisure words	Leisure	Singing, relaxing, vacation
LIWC Linguistic Clues		Family words	Home	House, family, pets
LIWC Linguistic Clues		Money words	Money	Rich, annual salary, discount
LIWC Linguistic Clues		Religious words	Religion	God, mercy, faith
LIWC Linguistic Clues		Death words	Death	Death, suicide, will
LIWC Linguistic Clues	Informal language	Should and word	Assent	Yes, really, good
LIWC Linguistic Clues		Pause filler	Nonfl	Well, then, that
LIWC Linguistic Clues		Filler filler	Filler	Just, like, say

LIWC Lingustic Clues		obscene language	Swear	Fuck you, retarded, idiot
Mental Health Topics	psychological problems	Depression and anxiety	Depression and anxiety	anxiety, insomnia, depression
Mental Health Topics	psychological problems	Discomfort	Discomfort	unhappy, sad, uncomfortable
Mental Health Topics	psychological problems	Social phobia	Social phobia	communication, self-abasement, introversion
Mental Health Topics	psychological problems	Lack of interest	Lack of interest	no interest, no drive, no confidence
Mental Health Topics	psychological problems	Suicidal tendency	Suicidal tendency	suicide, self-harm, tendency
Mental Health Topics	psychological problems	Fear	Fear	fear, worry, tension
Mental Health Topics	psychological problems	Angry	Angry	anger, dislike, tantrums
Mental Health Topics	Influencing factors	In Love/Boy or girl friend	In Love/Boy or girl friend	love, boyfriend, relationship
Mental Health Topics	Influencing factors	Marriage	Marriage	marriage, divorce, children

Mental Health Topics	Influencing factors	Psychotherapy	Psychotherapy	treatment, diagnosis, pandemic
Mental Health Topics	Influencing factors	Work	Work	job, graduation, resignation
Mental Health Topics	Influencing factors	Interpersonal relationship	Interpersonal relationship	communication, friend, contact
Mental Health Topics	Influencing factors	Personal characteristics	Personal characteristics	character, emotion, personality
Mental Health Topics	Influencing factors	Family	Family	parents, mother, family
COVID-19 related cases	\	Cumulative Confirmed Cases	cumulative confirmed cases	\
COVID-19 related cases	\	Cumulative Deaths	cumulative deaths	\
COVID-19 related cases	\	Newly Confirmed Cases	newly confirmed cases	\
COVID-19 related cases	\	New Deaths	new deaths	\

Facebook Prophet	\	Yearly Seasonality	yearly	\
Facebook Prophet	\	Weekly Seasonality	weekly	\
Facebook Prophet	\	General Trend	trend	\
Facebook Prophet	\	weekly plus yearly seasonality	additive terms	\
Facebook Prophet	\	predicted value	yhat	\
